# Effects of Docetaxel plus Degarelix on Quality of Life and Vascular Endothelial Growth Factor in Patients with Prostate Cancer

**DOI:** 10.1155/2022/9082501

**Published:** 2022-07-13

**Authors:** Weiping Li, Dongbo Xu, Fudong Li, Pengcheng Chang, Bin Zhang

**Affiliations:** ^1^Department of Urology, The First Hospital of Lanzhou University, Lanzhou, China; ^2^Department of Urology, The 940 Hospital of Joint Logistics Support Force of Chinese PLA, Lanzhou, China

## Abstract

**Objective:**

To investigate the effects of docetaxel plus degarelix on quality of life and vascular endothelial growth factor in patients with prostate cancer.

**Methods:**

Between 2018 and 2020, 38 patients with castration-resistant prostate cancer (CRPC) treated in our institution were assessed for eligibility and recruited. They were assigned at a ratio of 1 : 1 to receive either docetaxel plus degarelix (observation group) or degarelix (control group). Outcome measures included treatment efficacy, inflammatory factors level, vascular endothelial growth factor (VEGF) level, and quality of life of patients.

**Results:**

Docetaxel plus degarelix was associated with a significantly higher treatment efficacy (94.74%, including 9 (47.37%) cases of complete response (CR), 6 (31.58%) cases of partial response (PR), 4 (21.05%) cases of stable disease (SD), and 1 (5.36%) case of progressive disease (PD)) versus degarelix alone (63.16%, including 4 (21.05%) cases of CR, 5 (26.32%) cases of PR, 3 (15.79%) cases of SD, and 7 (36.84%) cases of PD) (*P* < 0.05). Before treatment, the two groups showed comparable levels of C-reaction protein (CRP), interleukin- (IL-) 6, and IL-10 (*P* > 0.05). Docetaxel plus degarelix resulted in significantly reduced levels of CRP and IL-6 and a significantly higher IL-10 level (28.84 ± 5.42, 25.31 ± 5.74, and 53.32 ± 11.02) versus degarelix alone (35.17 ± 6.31, 31.54 ± 8.17, and 42.76 ± 11.25) (*P* < 0.05). There were no significant differences in the urinary function, intestinal function, and hormone function scores between the two groups before treatment (*P* > 0.05). The patients receiving docetaxel plus degarelix had higher urinary function, intestinal function, and hormone function scores (38.87 ± 4.46, 86.51 ± 8.14, and 76.65 ± 7.15) versus monotherapy of degarelix (29.84 ± 3.58, 78.51 ± 7.31, and 66.78 ± 6.56) (*P* < 0.05). The two groups had similar pretreatment VEGF levels (*P* > 0.05). Docetaxel plus degarelix resulted in significantly lower VEGF levels (119.17 ± 21.38) versus degarelix (124.36 ± 23.14) at 6 months after treatment (*P* < 0.05).

**Conclusion:**

Docetaxel plus degarelix can enhance the therapeutic efficacy of patients with prostate cancer, mitigate inflammatory response, inhibit the VEGF expression of cancer cells, and improve the patients' quality of life. Further clinical trials are, however, required prior to general use in clinical practice.

## 1. Introduction

Prostate cancer is a tumor due to the malignant proliferation of epithelial cells in the prostate [1], mainly including squamous cell carcinoma and prostate adenocarcinoma [2]. It is characterized by high prevalence, high mortality rate, and poor prognosis [3]. The incidence of prostate cancer in China was 9.92/100,000 in 2012, accounting for the sixth most common malignant tumor in males [4], and it is most prevalent in people aged 70-80 years. The primary clinical treatments for prostate cancer are surgery and hormonal therapy. The early symptoms of prostate cancer are insidious [5], and it has been reported [6] that about 20% of prostate cancer cases in China experience distant metastases at the time of clinical diagnosis. Several studies have proposed [7, 8] the use of docetaxel for the treatment of metastatic prostate cancer, advanced carcinoma in situ, hormone-sensitive prostate cancer, and chemoresistant prostate cancer, and the Chinese Guidelines for the Diagnosis and Treatment of Urological Diseases: 2019 edition recommends docetaxel-based therapy as the standard treatment option for desmoid-resistant prostate cancer [9], Degarelix is commonly used in the endocrine treatment of prostate cancer. It regulates the secretion of testosterone through the hypothalamic-pituitary-gonadal axis [10] and reduces serum testosterone levels, thereby inhibiting cancer cell growth. However, the drug is associated with various adverse events such as negative emotions, decreased libido, cognitive dysfunction, and erectile dysfunction [11]. Degarelix is used in the debulking treatment of advanced prostate cancer, primarily by inhibiting the release of testosterone from the testes, thereby suppressing the progression of prostate cancer. Degarelix has been shown to be effective in reducing plasma testosterone levels and tumor growth rates at levels comparable to those after surgical debulking. Compared to GnRHa, the use of degarelix has a pharmacological profile closer to that seen during orchiectomy, with faster testosterone and prostate-specific antigen inhibition and no testosterone surge or microwave motility. Thus, there is no risk of clinical flare-ups with its use and no need for concomitant antiandrogen surge protection. Currently, the combination of degarelix and docetaxel in the treatment of prostate cancer has been marginally explored. Accordingly, this study was conducted to investigate the effects of docetaxel plus degarelix on quality of life and vascular endothelial growth factor in patients with prostate cancer.

## 2. Materials and Methods

### 2.1. Participants

Between 2018 and 2020, 38 patients with castration-resistant prostate cancer (CRPC) treated in our institution were assessed for eligibility and recruited. They were assigned at a ratio of 1 : 1 to either an observation group or a control group. The baseline characteristics of the observation group (aged 46-79 years, mean age of 45.85 ± 5.78 years, duration of disease of 2.5-3.2 years, mean duration of disease of 3.18 ± 0.48 years, and 11 cases of grade 3 and 8 cases of grade 4/5 in terms of Gleason pathological grading) were comparable with those of the control group (aged 46-80 years, mean age of 45.79 ± 6.02 years, duration of disease of 2.8-3.4 years, mean duration of disease of 3.07 ± 0.39 years, 12 cases of grade 3 and 7 cases of grade 4/5 in terms of Gleason pathological grading) (*P* > 0.05).(Table 1) (Gleason grading: grade 1: uniformly regular large glands were densely and dorsally packed to form small nodules; grade 2: more irregular large glands weredensely and dorsally packed to form small nodules, with nonfused glands within the nodules; grade 3: small infiltrating growing glands or vesicles or small sieve-like structured glands were seen; grade 4: fused glands, large sieve glands, or a renal clear cell carcinoma-like appearance were seen; grade 5: solid carcinoma nests were seen with a single cancer cell infiltration, or a pimple-like carcinoma). The study was approved by the First Hospital of Lanzhou University, and the ethical certificate number of this study is 2017-11-19.

### 2.2. Inclusion and Exclusion Criteria

Patients who were diagnosed with mCRPC [4] and with consciousness and no comorbid psychiatric disorders were included. Patients and family members were informed about this study and provided written informed consent.

Patients with immune system and respiratory system diseases with an expected survival of <1 year and with coagulation dysfunctions were excluded.

### 2.3. Treatment Methods

Patients in the control group were treated with degarelix (AstraZeneca UK limited, approval no. J20100126) for two months with subcutaneous injections every 4 weeks at a dose of 3.6 mg. The prostate-specific antigen level (PSA) was examinedevery month, and the treatment was continued at PSA > 4.0 ng/mL and was discontinued at PSA < 0.2 ng/mL. A similar administration regimen of degarelix was introduced to the patients in the observation group.

Patients in the observation group received docetaxel plus degarelix for two months. Docetaxel (Beijing Dongfang Xiehe Pharmaceutical and Biological Company, approval no. H20050879) was administered intravenously once every 28 days at a dose of 60-75 mg/m^2^. In the event of adverse reactions (e.g., vomiting, alopecia, anorexia, decreased white blood cells, and decreased immunity), the dose was reduced or discontinued as appropriate for severe reactions, and follow-up treatment was determined according to the patient's actual status.

### 2.4. Outcome Measures

1. Treatment efficacy: the efficacy was determined based on PSA outcomes. Complete response (CR): symptoms were improved significantly, PSA decreased by 50%, and quality of life was improved significantly. Partial response (PR): symptoms were alleviated, PSA decreased by <40%, and the quality of life was improved. Stable disease (SD): symptoms showed some alleviation, PSA decreased by 40-50%, and quality of life was improved. Progressive disease (PD): the above criteria were not met. Total efficacy = (CR + SD + PR)/total number of cases × 100%
2. Inflammatory factors: before and after the intervention, 3 mL of morning fasting venous blood was collected from the patients, rested for 30 min, and centrifuged at 2000 r/min for 15 min with a radius of 15 cm, and the serum was separated and stored in Eppendorf tubes in a medical refrigerator for assays. The levels of C-reactive protein (CRP) were determined by the immunohybrid assay, and the levels of interleukin-6 (IL-6) and interleukin-10 (IL-10) were determined by the enzyme-linked immunosorbent assay
3. Quality of life: the expanded prostate cancer index composite (EPIC) was used to assess the patients' quality of life before and after treatment. The scale entries A-E corresponded to a score of 0-4 points, respectively, and if only four options A-D were available, they corresponded to 0, 1, 2, and 4 points, respectively, with a total score of 0-100 points
4. Vascular endothelial growth factor (VEGF): the levels of VEGF were determined by enzyme-linked immunosorbent assay before and at 1, 3, and 6 months after the intervention, respectively

### 2.5. Statistical Analysis

SPSS 22.0 was used for data analyses. The measurement data are expressed as (mean ± SD) and analyzed using the *t*-test. The count data are expressed as the number of cases (rate) and analyzed using the chi-square test. Differences were considered statistically significant at *P* < 0.05.

## 3. Results

### 3.1. Clinical Efficacy

Docetaxel plus degarelix was associated with a significantly higher treatment efficacy (94.74%, including 9 (47.37%) cases of CR, 6 (31.58%) cases of PR, 4 (21.05%) cases of SD, and 1 (5.36%) case of PD) versus degarelix alone (63.16%, including 4 (21.05%) cases of CR, 5 (26.32%) cases of PR, 3 (15.79%) cases of SD, and 7 (36.84%) cases of PD) (*P* < 0.05) (Table 2).

### 3.2. Inflammatory Factors

Before treatment, the two groups showed similar levels of CRP, IL-6, and IL-10 (*P* > 0.05). Docetaxel plus degarelix resulted in significantly reduced levels of CRP and IL-6 and a significantly higher IL-10 level (28.84 ± 5.42, 25.31 ± 5.74, and 53.32 ± 11.02) versus degarelix alone (35.17 ± 6.31, 31.54 ± 8.17, and 42.76 ± 11.25) (*P* < 0.05) (Table 3).

### 3.3. Quality of Life

No significant differences were found in the urinary function, intestinal function, and hormone function scores between the two groups before treatment (*P* > 0.05). The patients receiving docetaxel plus degarelix showed higher urinary function, intestinal function, and hormone function scores (38.87 ± 4.46, 86.51 ± 8.14, and 76.65 ± 7.15) versus monotherapy of degarelix (29.84 ± 3.58, 78.51 ± 7.31, and 66.78 ± 6.56) (*P* < 0.05) (Table 4).

### 3.4. Vascular Endothelial Growth Factor

The two groups had similar pretreatment VEGF levels (*P* > 0.05). Docetaxel plus degarelix resulted in significantly lower VEGF levels (119.17 ± 21.38) versus degarelix (124.36 ± 23.14) at 6 months after treatment (*P* < 0.05). At 1 and 3 months after treatment, no intergroup significant differences were seen (*P* > 0.05) (Table 5).

## 4. Discussion

Prostate cancer is a public health issue of wide global concern [12]. Currently, clinical treatment is surgery and hormone therapy. Docetaxel is a semisynthetic paclitaxel antitumor analog [13] with strong antitumor activity effects, downregulates gene expression during tumor development [14], and provides therapeutic effects by inhibition of androgen receptors. However, the cytotoxicity of docetaxel may affect granulocytes, leading to intolerance, myelosuppression, gastrointestinal reactions, fluid retention, and angioedema [15, 16]. Degarelix is a drug commonly used in the endocrine treatment of prostate cancer [17], which regulates the secretion of testosterone through the hypothalamic-pituitary-gonadal axis and reduces serum testosterone levels, thereby inhibiting cancer cell growth [18], but it is also associated with multiple adverse events such as negative emotions, decreased libido, cognitive dysfunction, and erectile dysfunction in patients [19, 20]. The prolonged effect of degarelix is because the subcutaneous injection of degarelix creates an “in situ depot” from which degarelix is slowly released into the body circulation to exert its pharmacological effects. The nature of depot has not been determined, but it seems to contain a gel structure that presumably forms once in contact with degarelix, for example in histones after administration, and then, the active compound (degarelix) is slowly released from the depot. This particular depot is an advantage in the treatment of prostate cancer because the effective plasma concentration can be maintained for a long duration [19]. Doxorubicin is a chemotherapy drug approved by the FDA for the treatment of prostate cancer. Mitoxantrone and fosfestrol have failed to prolong the survival of patients, although they can improve the quality of life and reduce the serum prostate-specific antigen compared with hormones alone. Doxorubicin, however, is the only chemotherapeutic agent that has been shown to prolong the survival of patients with prostate cancer.

In the present study, docetaxel plus degarelix was associated with significantly higher treatment efficacy and significantly reduced levels of CRP and IL-6 and a significantly higher IL-10 levelversus degarelix alone. The reason may be that degarelix decreases insulin sensitivity, while low-density lipoprotein (LDL) and triglyceride levels rise after being affected, and docetaxel is an antitumor drug that uses microtubules as a target and blocks cancer cells in the M phase, thus effectively mitigating the inflammatory response and enhancing the therapeutic effect, which is consistent with the results in previous clinical studies. The combination of degarelix and docetaxel can achieve a synergistic effect to reduce serum PSA levels, mitigate the adverse events elicited by endocrine therapy, and improve the quality of life of patients. Here, the patients receiving docetaxel plus degarelix showed higher urinary function, intestinal function, and hormone function scores versus monotherapy of degarelix. Degarelix plus docetaxel facilitates the amelioration of urinary tract symptoms and gastrointestinal symptoms to enhance patients' quality of life. Blood metastasis is a major pathway for the spread of prostate cancer, and VEGF, as a cell growth factor with multiple functions, acts specifically on vascular endothelial cells and accelerates tumor angiogenesis. The results of the present study showed that docetaxel plus degarelix resulted in significantly lower VEGF levels versus degarelix at 6 months after treatment, which may be attributed to that docetaxel can downregulate genes associated with tumor development and inhibit tumor blood vessel growth, thereby preventing transvascular metastasis of tumor cells.

The combination of doxorubicin and prednisone has shown a significant clinical effect on HRPC. Doxorubicin plus osteopontin can inhibit the growth of prostate cancer cells through vDR receptor action in multiple pathways, including inhibition of proliferation, mediation of apoptosis, promotion of differentiation, and reduction of cell infiltration. In the regimen of doxorubicin plus estramustine phosphate, in which estramustine phosphate is a nitrogen mustard antitumor compound with estradiol phospholipid as the carrier, with the dual action of nitrogen mustard and estrogen, high affinity for prostate, lower tumor suppression rate but light estrogenic effect compared with estradiol. In the doxorubicin combined with ifosfamide regimen, ifosfamide is an alkylated oxazaphosphate ring drug structurally similar to cyclophosphophthalamide. It is a precursor drug that is converted to a cytotoxically active metabolite by enzymatic action in the liver. Its mechanism of action is similar to that of other alkylating agents, as it interferes with DNA synthesis by irreversibly cross-linking with DNA strands.

To sum up, docetaxel plus degarelix can enhance the therapeutic efficacy of patients with prostate cancer, mitigate inflammatory response, inhibit the VEGF expression of cancer cells, and improve the patients' quality of life. Further clinical trials are, however, required prior to general use in clinical practice.

## Figures and Tables

**Table 1:** Comparison of baseline data ($\bar{x}\pm s$).

Groups	*n*	Age	Mean age	Duration of disease	Mean duration of disease	Gleason grading	
						Grade 3	Grade 4/5
Observation group	19	46-79	45.85 ± 5.78	2.5-3.2	3.18 ± 0.48	11	8
Control group	19	46-80	45.79 ± 6.02	2.83.4	3.07 ± 0.39	12	7
*t* value	—	—	0.051	—	0.915	—	—
*P* value	—	—	0.959	—	0.362	—	—

**Table 2:** Comparison of clinical efficacy (%).

Groups	*n*	CR	SD	PR	PD	Total efficacy
Observation group	19	9 (47.37)	6 (31.58)	4 (21.05)	1 (5.36)	18 (94.74)
Control group	19	4 (21.05)	5 (26.32)	3 (15.79)	7 (36.84)	12 (63.16)
*x* ^2^	—	—	—	—	—	14.62
*P* value	—	—	—	—	—	<0.001

Note: CR: complete response; PR: partial response; SD: stable disease; PD: progressive disease.

**Table 3:** Comparison of inflammatory factors levels ($\bar{x}\pm s$).

Groups	Timepoints	Observation group (*n* = 19)	Control group (*n* = 19)	*t* value	*P* value
CRP	Before treatment	38.02 ± 6.23	37.98 ± 6.39	0.032	0.975
	2 months after treatment	28.84 ± 5.42	35.17 ± 6.31	5.381	<0.001

IL-6	Before treatment	35.91 ± 7.83	35.84 ± 7.96	0.044	0.965
	2 months after treatment	25.31 ± 5.74	31.54 ± 8.17	4.412	<0.001

IL-10	Before treatment	36.37 ± 10.21	36.48 ± 10.03	0.054	0.975
	2 months after treatment	53.32 ± 11.02	42.76 ± 11.25	4.742	<0.001

**Table 4:** Comparison of quality of life ($\bar{x}\pm s$).

Groups	Timepoints	Observation group (*n* = 19)	Control group (*n* = 19)	*t* value	*P* value
Urinary function	Before treatment	52.12 ± 4.12	52.85 ± 3.68	0.934	0.353
	After treatment	38.87 ± 4.46	29.84 ± 3.58	11.165	<0.001

Intestinal function	Before treatment	95.27 ± 2.48	94.96 ± 3.05	0.558	0.578
	After treatment	86.51 ± 8.14	78.51 ± 7.31	5.171	<0.001

Hormone function	Before treatment	85.79 ± 8.24	84.68 ± 8.97	0.644	0.521
	After treatment	76.65 ± 7.15	66.78 ± 6.56	7.192	<0.001

**Table 5:** Comparison of vascular endothelial growth factor levels ($\bar{x}\pm s$).

Groups	*n*	Before treatment	1 month after treatment	3 months after treatment	6 months after treatment
Observation group	19	461.21 ± 91.98	372.14 ± 72.39	298.15 ± 63.58	118.01 ± 21.01
Control group	19	461.34 ± 92.08	365.56 ± 73.65	284.99 ± 60.37	125.98 ± 19.88
*t*	—	0.007	0.451	1.061	1.989
*P*	—	0.994	0.653	0.291	0.049

## Data Availability

All data generated or analyzed during this study are included in this published article.
